# An old disease with a new twist: CNS and thyroid sarcoidosis presenting as subacute dementia

**DOI:** 10.1002/ccr3.4829

**Published:** 2021-10-06

**Authors:** Brooke Pace Quertermous, Sravan Kavuri, David W. Walsh

**Affiliations:** ^1^ Medical College of Georgia Augusta University Augusta GA USA

**Keywords:** dementia, hyperthyroidism, sarcoidosis

## Abstract

Dementia in the elderly is extremely common and is often irreversible. When a patient presents with rapid cognitive decline, uncommon reversible etiologies should be investigated with the goal of restoring cognitive function.

## Graphical abstract

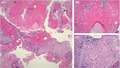

## 1 INTRODUCTION

Neurosarcoidosis and thyroid abnormalities are reversible causes of dementia in the elderly. We present a case of a patient with rapidly progressive dementia due to neurosarcoidosis and sarcoid involvement of the thyroid improved by corticosteroids and methimazole.

Systemic sarcoidosis is an inflammatory condition characterized by the presence of non‐caseating granulomas found in multiple organ systems. While the underlying pathophysiology has not been fully elucidated, autoimmunity appears to play a central role. As is common with autoimmune diseases, most patients are diagnosed between ages of 20 and 40. While almost any organ can be involved, the lungs and intrathoracic lymph nodes are the primary organs affected in over 90% of patients. Neurologic involvement occurs in approximately 5% of patients and may be the presenting symptom. These neurologic manifestations are collectively known as neurosarcoidosis which can involve both the brain and the spinal cord. Symptoms of cranial involvement include headache, visual impairment, and other signs of increased intracranial pressure. Other neurologic findings such as cranial nerve dysfunction, ataxia, aphasia, and seizure can be present, as well as mood symptoms such as depression and cognitive impairment or dementia. Granulomatous invasion of the spinal cord can present as myelopathy, peripheral neuropathy, or myopathy, earning neurosarcoidosis the nickname “the great mimicker” by many neurologists.^1^ The thyroid is a less commonly affected organ, with less than 70 cases of clinical thyroid involvement due to systemic sarcoidosis reported in the literature. However, granulomatous inflammation of the thyroid due to systemic sarcoidosis has been described as a unique and presenting localization of disease with no evidence of pulmonary or other extrapulmonary manifestations.^2^ Hypothyroidism or hyperthyroidism can be present, and there is an association between systemic sarcoidosis and autoimmune thyroid pathologies such as Hashimoto’s thyroiditis and Grave’s disease.^3^

## 2 CASE HISTORY/EXAMINATION

A 64‐year‐old African‐American female patient with a past medical history of hypertension and vitamin D deficiency presented to our emergency department from home following a witnessed syncopal episode. She denied headaches, vision changes, chest pain, palpitations, and dyspnea. Since retirement one year ago, her family has noticed progressive functional decline. They note a more rapid decline over the past 6 months and report that she struggles with memory loss and maintaining her activities of daily living. On initial evaluation, the patient had a regular heart rate, normal rhythm, and no murmurs or additional heart sounds were appreciated; lungs were clear to auscultation bilaterally. Neurologic examination revealed no focal neurologic sensory or motor deficits; however, patient relied heavily on caregiver to relay information to the provider and had waxing and waning level of consciousness. She also displayed global decrease in strength, without any noted muscle wasting. Sit‐to‐stand vitals were performed and showed no evidence of orthostasis.

## 3 DIFFERENTIAL DIAGNOSIS, INVESTIGATION, AND TREATMENT

**FIGURE 1:**
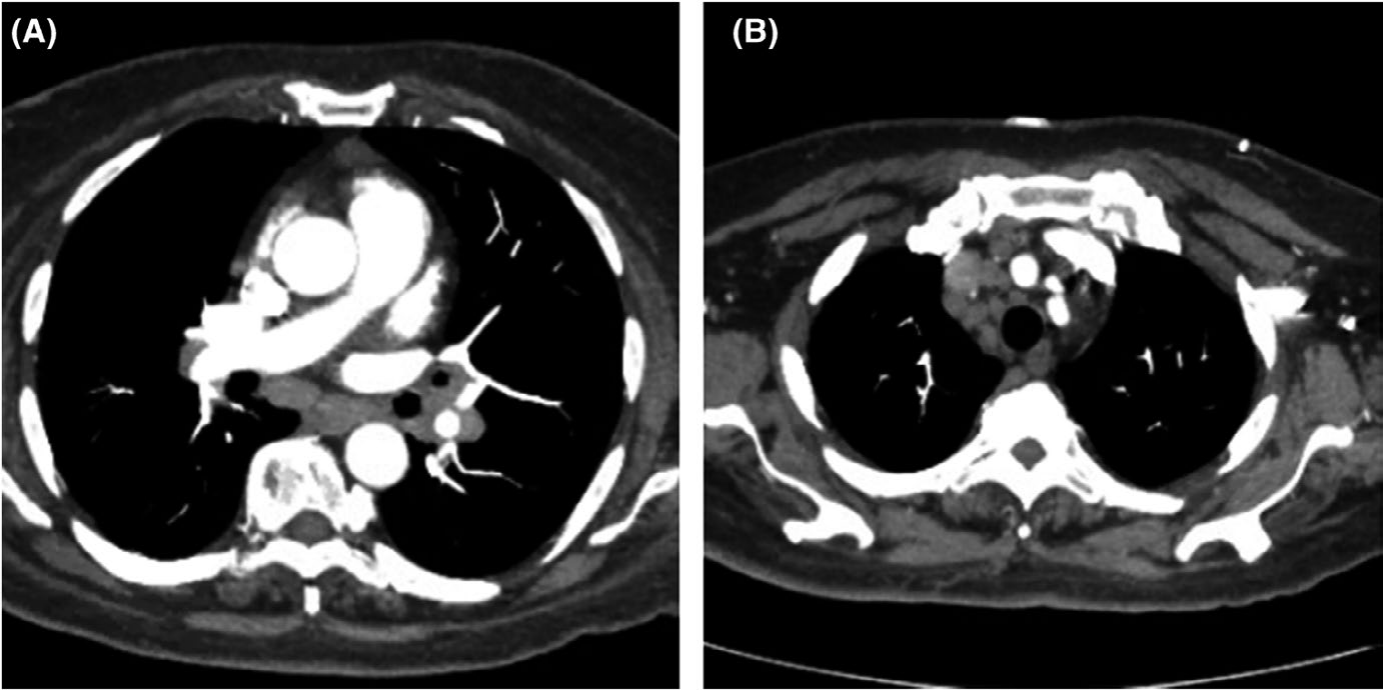
Computed tomography angiography (CT) of the chest showing new‐onset mediastinal (A) and hilar (B) lymphadenopathy

**FIGURE 2:**
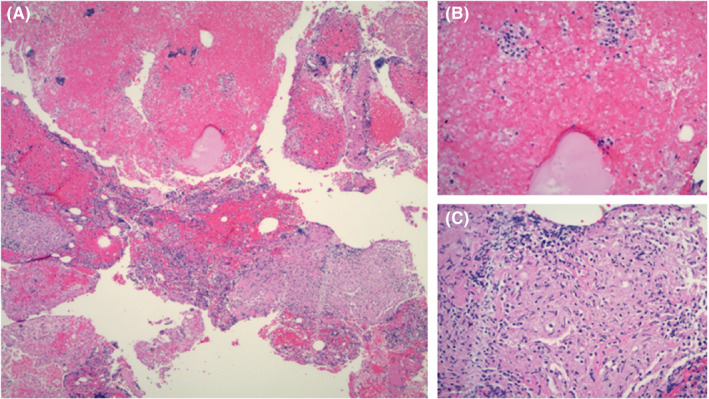
Pathology of thyroid biopsy using hematoxylin‐eosin (HE) staining. A, Low power view (4×) with granulomas, colloid, and follicular groups. B, High power view (20×) with colloid and follicular groups. C, High power view with multiple granulomas

A syncope workup was initiated and unremarkable aside for low thyroid‐stimulating hormone (TSH, 0.317 mcIU/ml) and high thyroid hormone (T4 12.7 mcg/dl; Free T4 1.84 ng/dl). Calcium corrected for albumin was increased (13.3 mg/dl) and 25‐OH Vitamin D was low (22.63 ng/ml). Incidentally, chest computed tomography (CT) revealed extensive mediastinal and hilar lymphadenopathy (Figure 1) along with an enlarged thyroid. These findings were new onset since the patient’s last chest CT before 7 weeks. A tuberculin skin test was placed and was not reactive. The leading differential diagnoses at this point included 1) neoplasm of the lung or lymphatic system due to intrathoracic lymphadenopathy, and 2) pulmonary sarcoidosis due to lymphadenopathy with coexisting hypercalcemia and hypovitaminosis D. Endobronchial biopsy was performed and revealed non‐caseating granulomas in the hilar and mediastinal lymph nodes; no evidence of malignancy was found, and a diagnosis of pulmonary sarcoidosis was suggested. Intraoperatively, core needle biopsy specimens of the thyroid were also collected due to the goiter seen on CT and revealed non‐caseating granulomas in the thyroid tissue (Figure 2). Further thyroid labs were obtained, and thyroid‐stimulating immunoglobulin (TSIg) and anti‐thyroid peroxidase (anti‐TPO) antibodies were unremarkable, ruling out Graves’ disease and Hashimoto’s thyroiditis. The patient’s thyroglobulin level was 393 ng/mL, almost ten times the upper limit of normal (3–40 ng/ml). Endocrinology was consulted and the patient was started on methimazole for subacute thyrotoxicosis. Neurology was consulted for evaluation of neurosarcoidosis. An MRI of the brain showed no definitive evidence of sarcoid involvement. A series of lumbar punctures were performed which showed low glucose, high protein, and 19 leukocytes; the differential was then narrowed to atypical bacterial or fungal meningitis versus neurosarcoidosis. Tests for Lyme disease and histoplasmosis using cerebrospinal fluid (CSF) were negative, beta‐D glucan was not present, but angiotensin‐converting enzyme (ACE) was elevated (3.5 U/L). MRI of cervical and thoracic spine showed nodular leptomeningeal enhancement at the dorsal cervicomedullary junction and conus medullaris, consistent with neurosarcoidosis. Due to these imaging findings, elevated ACE in the CSF, and progressive, chronic neurologic decline for the past 6 months, this patient’s presentation with recurrent syncope and progressive dementia can be attributed to neurosarcoidosis with superimposed metabolic encephalopathy likely due to hyperthyroidism caused by sarcoid involvement of the thyroid gland.

## 4 OUTCOMES AND FOLLOW‐UP

The patient was started on high‐dose oral prednisone and was discharged to a subacute rehabilitation facility for upon improvement. She remained in the rehabilitation facility for 2 weeks and was then discharged. At her neurology follow‐up 6 weeks later, the patient and her family noted marked clinical improvement.

## 5 DISCUSSION

To our knowledge, there are no reports of neurosarcoidosis with coexisting sarcoid involvement of the thyroid gland contributing to acute chronic cognitive decline. This patient presented with a progressive decline in cognitive function for the past 6 months that impacted her memory and her ability to care for herself. It has been well‐documented that both neurosarcoidosis and hyperthyroidism can contribute to dementia. The mechanism by which neurosarcoidosis affects the brain is more overt, as granuloma formation within the brain can disrupt vital brain functioning.^4^ Neurosarcoidosis has also been described as a cause of reversible rapidly progressive dementia (RPD), which is defined as cognitive decline progressing to severe cognitive impairment, often requiring assistance to maintain activities of daily living, normally occurring within weeks to months.^5^ Additionally, hypercalcemia caused by sarcoidosis can contribute to altered mental status. Psychiatric symptoms of hypercalcemia include lethargy, confusion, depression, and memory loss.^6^ The pathophysiology of hyperthyroidism contributing to dementia, however, is not so clear. Several mechanisms have been proposed including reduced clearance of amyloid‐beta plaques in patients with elevated thyroid hormone^7^ and increased oxidative stress in hyperthyroid patients leading to vascular and neuronal degeneration in the brain.^8^ Some studies have shown that treating hyperthyroidism can lead to partial or complete reversal of dementia symptoms^9^ Therefore, treatment of neurosarcoidosis and hyperthyroidism in this patient was imperative as adequate control of disease may restore cognition.

Neurosarcoidosis is usually treated with high‐dose glucocorticoids with progressive tapering as symptoms improve. Treatment of hyperthyroidism is a twofold approach aimed treating adrenergic symptoms and preventing excess thyroid hormone production. Adrenergic symptoms are most effectively treated with a beta‐blocker, and thyroid hormone production can be decreased using anti‐thyroid agents such as methimazole, propylthiouracil, and radioactive iodine. However, methimazole and propylthiouracil can cause agranulocytosis and should be used with caution and frequent monitoring in patients on other immunosuppressive therapies, such as our patient. Interestingly, glucocorticoids have also been shown to reduce conversion of T4 to the more active T3 and can be used for prevention of thyroid storms in patients with severe hyperthyroidism.^10^

As discussed previously, the clinical manifestations of systemic sarcoidosis are extremely variable from patient to patient. Our patient presented with pulmonary, central nervous system, and endocrine involvement. There is no scale to approximate the severity of extrapulmonary sarcoidosis; however, due to the involvement of two less commonly affected organ systems and the presence of rapidly progressive dementia, we can conclude that this patient has a very unique, widespread manifestation of disease. Interestingly, 7 weeks before her presentation to our emergency department and subsequent diagnosis of sarcoidosis, CT imaging to rule out upper‐gastrointestinal bleed noted only one enlarged epicardiac lymph node and no evidence of thyroid enlargement. When she arrived in our emergency department less than two months later, the radiologist noted extensive hilar and mediastinal lymphadenopathy and an abnormal thyroid gland, suggesting a rapid progression of her disease. This patient was also found to have an extremely elevated thyroglobulin level. Thyroglobulin (Tg) can be used as a tumor marker in patients who have undergone total thyroidectomy for thyroid cancer; however, in patients with thyroid, the American Thyroid Association does not support the use of serum Tg to screen for or detect thyroid cancer. Because of the strong association between elevated serum thyroglobulin and thyroid cancer, this patient will receive comprehensive endocrinology evaluation. In 2019, Hu et al described regulatory T cell dysfunction in patients with elevated thyroglobulin levels.^11^ Impairment of regulatory T cells is one of the leading proposed mechanisms for granuloma formation and maintenance in sarcoidosis.^12^ Immunologic studies have demonstrated that among patients with sarcoidosis, T‐regulatory cells fail to inhibit cytokines responsible for granuloma formation such as TNF‐ α, INF‐ γ, and IL‐2.^13^, ^14^ This patient’s extreme elevation in thyroglobulin combined with inherent T‐regulatory cell dysfunction in sarcoidosis likely contributed to her rapid disease progression. This patient’s manifestation of disease with rapid progression and elevated thyroglobulin also supports the argument of sarcoidosis as an autoimmune disease. In conclusion, we report a rare manifestation of systemic sarcoidosis involving the central nervous system and the thyroid gland contributing to a clinical picture of rapidly progressive dementia. This patient’s disease is likely reversible with treatment aimed at suppressing the immune system and limiting the production of excess thyroid hormone. It is very likely that elevated thyroglobulin contributed to the rapidly progressive course of disease in this patient by further inhibiting dysfunctional T‐regulatory cells found in patients with systemic sarcoidosis.

## CONFLICTS OF INTEREST

The authors have no conflicts of interest to declare.

## AUTHOR CONTRIBUTIONS

BQ and DW cared for the patient, conducted research, and prepared the manuscript. SK prepared and analyzed pathology specimen and provided clinical images.

### ETHICAL APPROVAL

The patient and her caregiver provided written consent to publish this report, including the publication of images.

### CONSENT

Published with written consent of the patient.

## Data Availability

Data sharing not applicable to this article as no datasets were generated or analysed during the current study.
